# Associations of streptococci and fungi amounts in the oral cavity with nutritional and oral health status in institutionalized elders: a cross sectional study

**DOI:** 10.1186/s12903-021-01926-0

**Published:** 2021-11-19

**Authors:** Hanako Sato, Akira Yano, Yu Shimoyama, Toshiro Sato, Yukiko Sugiyama, Mitsuo Kishi

**Affiliations:** 1 Division of Preventive Dentistry, Department of Oral Medicine, Iwate Medical University School of Dentistry, 1-3-27 Chuo-dori, Morioka, Iwate 020-8505 Japan; 2 Iwate Biotechnology Research Center, 174-4 Narita 22 Jiwari, Kitakami, Iwate 024-0003 Japan; 3 Division of Molecular Microbiology, Department of Microbiology, Iwate Medical University, 1-1 Idai Dori 1 chome, Yahaba, Iwate 028-3694 Japan

**Keywords:** Body mass, Oral health, Elder, Streptococci, Fungi

## Abstract

**Background:**

Disruption of the indigenous microbiota is likely related to frailty caused by undernutrition. However, the relationship between undernutrition and the oral microbiota, especially normal bacteria, is not obvious. The aim of this study was to elucidate the associations of nutritional and oral health conditions with prevalence of bacteria and fungi in the oral cavity of older individuals.

**Methods:**

Forty-one institutionalized older individuals with an average age ± standard deviation of 84.6 ± 8.3 years were enrolled as participants. Body mass index (BMI) and oral health assessment tool (OHAT) scores were used to represent nutritional and oral health status. Amounts of total bacteria, streptococci, and fungi in oral specimens collected from the tongue dorsum were determined by quantitative polymerase chain reaction (PCR) assay results. This study followed the STROBE statement for reports of observational studies.

**Results:**

There was a significant correlation between BMI and streptococcal amount (ρ = 0.526, *p* < 0.001). The undernutrition group (BMI < 20) showed a significantly lower average number of oral streptococci (*p* = 0.003). In logistic regression models, streptococcal amount was a significant variable accounting for “not undernutrition” [odds ratio 5.68, 95% confidential interval (CI) 1.64–19.7 (*p* = 0.06)]. On the other hand, participants with a poor oral health condition (OHAT ≥ 5) harbored significantly higher levels of fungi (*p* = 0.028).

**Conclusion:**

Oral streptococci were found to be associated with systemic nutritional condition and oral fungi with oral health condition. Thus, in order to understand the relationship of frailty with the oral microbiota in older individuals, it is necessary to examine oral indigenous bacteria as well as etiological microorganisms.

**Supplementary Information:**

The online version contains supplementary material available at 10.1186/s12903-021-01926-0.

## Background

Along with the general aging of society worldwide, frailty is gaining attention. This is a common geriatric syndrome that increases the risk of poor health outcomes, including falls, functional disability, hospitalization, and mortality [1–3]. Epidemiological studies have shown high proportions of elder individuals with a frailty phenotype (weakness, slowness, unintentional weight loss, exhaustion, low physical activity) [4, 5]. Since the cycle of frailty generally begins from chronic malnutrition and consequent weight loss [2], the health of the oral cavity, located at the start of the digestive tract, is likely related with a frail condition [6]. Previous studies have found relationships between malnutrition and poor oral health conditions in older individuals, in which dental disease status or oral function, including dental caries, periodontitis, tooth loss, and mastication, or swallowing disorders were mainly investigated as malnutrition risks [7–10]. In investigations that used a microbial approach, a relationship of the oral microbiome, consisting of not only pathogenic agents but also non-pathogenic indigenous bacteria, with nutritional status and oral health status has been demonstrated in older subjects [11, 12]. It has also been shown that the indigenous oral microbiota, composed of various microorganisms, plays an important role in maintaining homeostasis of oral and systemic health [13]. In the oral cavity, indigenous microorganisms such as streptococci grow and develop a suitable microbiota based on the environment provided by the host [14]. Once the microbiota is established, it helps to protect the host from invasion by pathogenic microorganisms and subsequent infection [15, 16]. Therefore, when a normal oral microbiota is disturbed, the population of pathogenic microorganisms is easily increased, which causes oral and systemic diseases [17]. On the other hand, the oral microbiota can be affected by changes in the oral environment as well as general conditions, such as aging, immunosuppression, and medication [18–20].

Recently, oral fungi have received focus as etiological microorganisms, especially in the elders, and it has been shown that older individuals are more prone to colonization by fungi due to such factors as decreased immune response, reduced salivary flow, and denture use [20–22]. *Candida* species, the most prevalent fungi found in the human oral cavity, are normal commensal and asymptomatic organisms in healthy conditions [23]. However, in frail older individuals or immunocompromised patients, it can overgrow in oropharyngeal or esophageal mucosa, which causes a burning sensation, taste disorder, severe mucositis, and/or dysphagia, resulting in poor nutrition [24, 25]. Furthermore, even with the absence of such oral symptoms, a relationship of general malnutrition with certain fungi in the oral cavity of elderly individuals has been shown [26].

Fungal growth in the oral cavity as well as systemic malnutrition are thought to be associated with an alteration in prevalence of oral indigenous bacteria. Oral dryness is a condition that can enhance fungal growth, though is not suitable for growth of indigenous oral bacteria such as oral streptococci. In addition, a decrease in oral intake of food, which is generally the start of the frailty cycle, can have a negative effect on growth of oral streptococci, whose main energy source is carbohydrates from staple foods [14, 27, 28]. Thus, deterioration of general health and nutritional status may disrupt the normal microbiota by increasing fungi and decreasing indigenous bacteria amounts. Such an oral microbiome composition may increase problems with the teeth or oral mucosa, causing adverse effects on systemic nutritional intake. We speculated that simultaneous measurements of oral fungal and streptococcus levels would be useful for determining systemic and oral frailty. Although detailed analyses of the oral microbiota have been reported [29, 30], few studies that investigated fungi and bacteria analyzed those in the same oral specimens, due to different gene targets associated with the molecular microbiological methods used.

In the present study, the amounts of fungi, total bacteria, and commensal streptococci in tongue samples of institutionalized older individuals were determined, and systemic and oral conditions were assessed. From those results, the relationships of systemic nutritional condition and oral health status with oral microbial prevalence were investigated.

## Methods

### Ethical considerations

The study was performed according to the principles of the Declaration of Helsinki and was approved by The Ethics Committee of Iwate Medical University School of Dentistry (approval no. 01340). Individuals with decision-making ability were given information regarding the study protocol and enrolled as participants after providing documentation indicating consent. For those who lacked ability to make such a decision due to cognitive decline, study protocol information documentation was given to a closely related family member, who provided consent for participation. Individuals who refused to participate in the study survey or undergo analysis after providing consent were subsequently excluded.

### Study design and participants

**Fig. 1:**
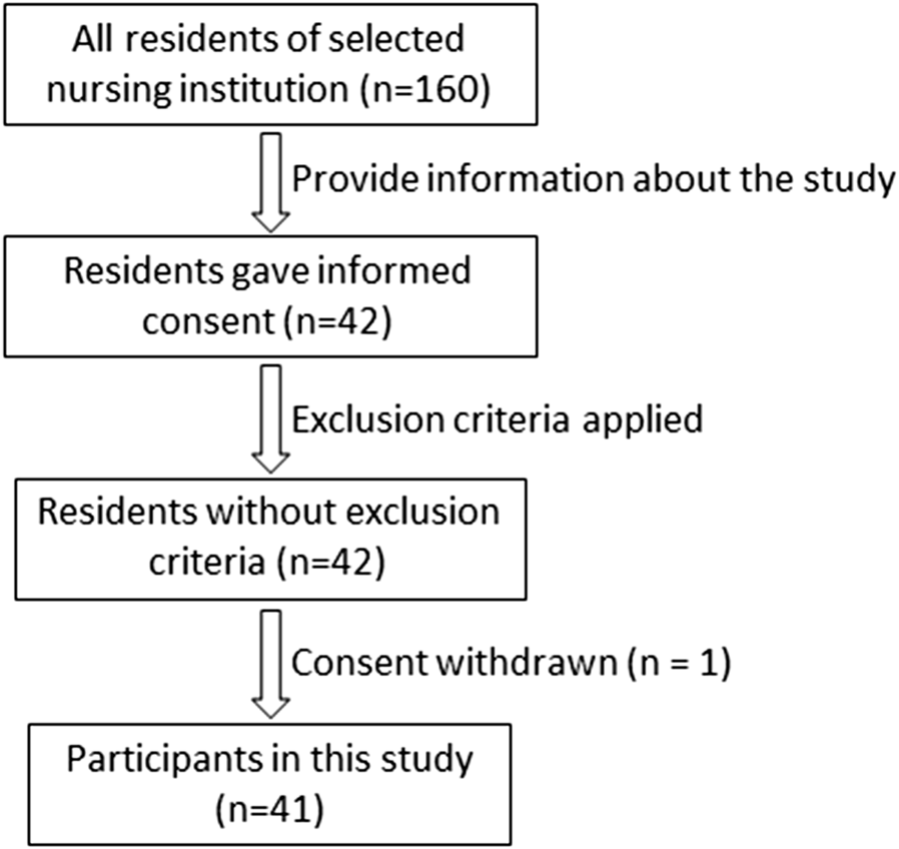
Flow diagram showing selection of the participants

This was a cross-sectional observational study and conducted at a nursing institution located in Iwate Prefecture, Japan. Informed consent was obtained from 42 of the 160 residents. Exclusion criteria included administration of antimicrobial or antifungal drugs within one month of the survey, as well as current status of undergoing parenteral nutrition. None of the 42 residents who provided consent to participate met those criteria. However, at the time of the oral examination, one rejected participation, after which none withdrew their consent. Consequently, 41 elder individuals (8 males, 33 females) with an average age ± standard deviation (SD) of 84.6 ± 8.3 years (range 70–105 years) completed the present study protocol (Fig. 1). None had a smoking or drinking habit. Two of the participants were undergoing special medical care, as one received dialysis treatment three times a week and the other wore a cardiac pacemaker, though neither presented outliers in regard to body mass index (BMI), oral health assessment tool (OHAT) results, or oral microbial amounts, and their results were included in the analyses. In addition, none suffered from oral cancer or an oral potentially malignant disorders (OPMD).

### Variables

The primary outcomes were amounts of oral streptococci, oral fungi, and total bacteria, which were determined using a quantitative polymerase chain reaction (PCR) method. The outcome used to represent systemic frailty was nutritional condition assessed by BMI, with that calculated from measurements conducted at the institution using the following formula: weight (kg)/height (cm)^2^. OHAT results were used for determination of oral frailty. To reveal relationships between those outcomes, information regarding potential confounders or effect modifiers, including gender, age, height, weight, application of special medical care, smoking and drinking habits, intake of antibacterial or anti-fungal medication, eating independence, and long-term care need level, was provided by the institution. This information was obtained from the facility care records, followed by confirmation with the caregiver in charge of each participant. Long-term care needs level is widely recognized in Japan as it is used in the national long-term care insurance system, which was established in 2000. Following receipt of an application from a community-dweller aged 65 years or older, their care needs level is determined by the applicable department for administration of their community based on examinations of mental and physical condition assessed by certification screening personnel, as well as a diagnosis from their primary physician. Long-term care needs consist of 7 levels, including support levels 1 and 2, and care levels 1–5, aside from not applicable. Support level 1 represents the lowest level of care needed and care level 5 the highest [31]. For the present study, the range from support level 1 to care level 5 is referred to as long-term care needs level 1–7. Differences in dietary habits were not considered in this study, because the facility provides nearly the same food menu to all residents for each meal service.

Potential confounders or effect modifiers concerning oral health were assessed by oral examinations performed by two well-calibrated dentists, including present number of teeth, present tooth status, presence of oral cancer or OPMD, tongue moisture level, tongue coating deposits, and denture use frequency. Both examiners had received training in oral assessment for perioperative care of inpatients at Iwate Medical University Hospital prior to the study.

### Oral examinations

Present tooth status including decayed and filled teeth was assessed according to the WHO criteria. Residual roots with or without coping treatment were recorded as a filled or decayed tooth, respectively. The presence of oral cancer or OPMD including leukoplakia, oral lichen planus, and erythroplakia, was determined using methods recommended by the WHO [32].

The moisture level of the tongue surface was measured using an intraoral moisture meter (Mucus®; Life, Saitama, Japan) at a point 1 cm posterior from the tip of the tongue along the median groove, as noted in a previous study [33]. Measurements were performed three times in each participant and the average value was used as the Mucus score. Tongue coating deposits were evaluated using tongue coating index (TCI). The surface of the tongue dorsum was divided into three sections vertically and three sections laterally (total nine sections). Each section was scored from 0 to 2 (0: tongue coating not visible, 1: thin tongue coating, papillae of tongue visible, 2: very thick tongue coating, papillae of tongue not visible) and the total score of the 9 sections was recorded as personal TCI [34]. Comprehensive oral health status was assessed using OHAT values, which is a validated tool used for assessment of oral health and comprises eight domains, including lips, tongue, gums and tissues, saliva, natural teeth, dentures, oral cleanliness, and dental pain, stratified into three grades (healthy, oral changes, unhealthy) [35]. In addition, at the oral examinations, denture use frequency was obtained by oral questioning of the participant or a caregiver. Denture use frequency was categorized into three levels; 1: no use, 2: used for meals, and 3: always worn except at sleeping time.

### Collection and genome purification of microbial samples

Microbial samples were collected immediately after the oral examination, as follows. The dorsum of the tongue was swabbed 20 times with a sterile cotton swab, which was then immersed in 1 ml of sterile saline. Collected samples were transferred to the laboratory on ice and stored at − 80 °C until genome extraction. Genomic DNA was extracted and purified from the collected samples using a Wizard® Genomic DNA Purification Kit (Promega), according to the manufacturer’s instructions. The microbial samples were lysed in 50 mM EDTA, 1 mg/ml lysozyme (Thermo Fisher Scientific, Waltham, USA), 0.5 mg/ml lysostaphin (Fujifilm Wako Pure Chemical), and 1 unit/ml lyticase (Sigma-Aldrich, St. Louis, MO, USA) at 37 °C for 30 min. In addition, samples were disturbed with an ISOFECAL for Beads Beating device (Nippon Gene, Tokyo, Japan) and a μT-12 beads crusher (TAITEC, Saitama, Japan) at 3200 r/minute for 5 min. Purified genomic DNA was dissolved in TE buffer (10 mM Tris–HCl, 1 mM EDTA, pH 8.0) and stored at − 20 °C.

### Primer sets for polymerase chain reaction (PCR) assays

A specific primer for amplifying the genome of oral streptococci was designed based on the *S. mutans* ATCC 25175 gene (NCBI Accession No. EF536028) and subjected to Primer-BLAST (basic local alignment search tool) (http://www.ncbi.nlm.nih.gov/tools/primer-blast/). The primer sequences (5′–3′) targeting all streptococci elongation factor-Tu were CCAATGCCACAAACTCGTGAAC (forward) and GATCACGGATTTCCATTTCAACC (reverse). To test the specificity of a streptococci-specific primer, the following strains were used: *Streptococcus mutans* ATCC 25175, *S. sobrinus* ATCC 27351, *S. salivarius* ATCC 7073, *S. oralis* ATCC 10557, *S. gordonii* ATCC 10558, *S. sanguinis* ATCC 10556, *S. mitis* ATCC 49456, *S. constellatus* ATCC 27823, *S. anginosus* NCTC10713, *S. intermedius* GAI 1157, *S. pyogenes* ATCC 12344, *Enterococcus faecalis* ATCC 29212, *Staphylococcus aureus* ATCC 25923, *Staph. epidermidis* ATCC 14990, *Fusobacterium nucleatum* ATCC 25586, and *Escherichia coli* BL21. They were cultured in trypticase soy broth (TSB, Becton Dickinson, Sparks, MD, USA) under an anaerobic condition (80% N_2_, 10% CO_2_, 10% H_2_) at 37 °C [36, 37].

One ng of each bacterial DNA sample was confirmed by detection with the streptococci specific primer set using KOD-Plus-Neo DNA polymerase (TOYOBO, Tokyo, Japan). The amplification cycles were as follows: 2 min at 98 °C for initial heat activation, then one cycle for 10 s at 94 °C, 10 s at 62 °C, and 5 s at 68 °C, for a total of 30 cycles. These PCR products were subjected to agarose gel electrophoresis using a 3.0% gel and stained with ethidium bromide, with detection performed at 302 nm with ChemiDoc XRS Plus (Bio-Rad Laboratories, Hercules, CA, USA). DNA amplicons were observed in ten oral streptococci, while no PCR products were demonstrated with DNA samples from the other six bacteria species (Additional file 1). For amplifying the genome of bacterial 16S rRNA universal and eukaryotic 18S rRNA genes, primer sets reported in previous studies were used [38, 39]. The primer sequences (5′–3′) targeting bacterial the 16S rRNA gene were CGCTAGTAATCGTGGATCAGAATG (forward) and TGTGACGGGCGGTGTGTA (reverse), and those (5′–3′) targeting the 18S rRNA gene were TCTCAGGCTCCYTCTCCGG (forward) and AAGCCATGCATGYCTAAGTATMA (reverse).

### Determination of microbial amounts

Stored samples were solved in room temperature. From those samples, genomic DNA was extracted and purified by the same method used for reference microorganisms. To determine microbial amounts in each sample, quantitative PCR assays were performed using a Thermal Cycler Dice Real-Time System II and with the following thermal cycle, as recommended for the TB Green Ex Taq (Takara Bio, Kusatsu, Japan) mixture: 95 °C for 30 s, then 40 cycles for 5 s at 95 °C and 1 m at 58 °C for 16S rRNA and 18S rRNA, and at 62 °C for oral streptococcal EF-Tu. Ten ng of genomic DNA from the samples was used as the template. Standard curves for each organism were plotted using *Ct* values obtained from amplification of genomic DNA. Those were extracted from *S*. *salivarius* ATCC 7073 (1.0 × 10^2^ to 1.0 × 10^6^ CFU) and *C. albicans* SC 5314 (1.0 × 10^1^ to 1.0 × 10^6^ CFU) cells. The numbers of *S*. *salivarius* and *C. albicans* were determined by plating culture dilutions in trypticase soy agar and YPD agar plates, respectively [38, 40]. The linearity of these assays was correlated between the *Ct* and microbial amounts. Correlation coefficients were 0.969 for 16S rRNA, 0.985 for oral streptococcal EF-Tu, and 0.979 for 18S rRNA. The amounts of the microorganisms are expressed as logarithm number of CFU.

### Statistical analysis

Age, BMI, care needs score, number of teeth, TCI, Mucus score, OHAT score, and amounts of oral microorganisms were used as continuous variables. After distributions of all continuous variables were tested by one-sample Kolmogorov–Smirnov test, variables with normal distribution (age, BMI, total bacterial amount, streptococcal amount) were used as quantitative variables. Variables that did not show a normal distribution treated as rank variables. Gender, need for special medical care, edentulous status, and denture wearer were treated as categorical variables.

For assessing nutritional condition, the participants were classified as BMI < 20 (undernutrition) or ≥ 20 (not undernutrition), according to the criteria for Asian elder people aged 70 years or older in The Global Leadership Initiative on Malnutrition (GLIM) [41]. For OHAT, the participants were divided into low (score ≤ 5) and high (score > 5) OHAT groups, after referring to the median of the participants in this study and a cut-off value in previous study [42]. For comparison tests between the groups, Mann–Whitney’s U test was used for rank variables and Fisher’s exact test for categorical variables. Single correlations for combinations of quantitative variables were tested using Pearson’s correlation coefficient analysis, while Spearman's rank correlation analysis was used for combinations of rank or categorical variables after the categorical variables were transformed into binary variables. Eating independence was entered as a three-level rank variable in Spearman's rank correlation analysis and as a categorical variable in the following comparisons between groups.

For multivariable analysis to elucidate factors related with BMI or OHAT, multiple logistic regression analyses were performed. In the model for BMI, the dependent variable was set as “not undernutrition” (BMI ≥ 20: 1, BMI < 20: 0) and three variables (streptococcal amount, eating independence level, TCI) were selected as independent variables based on the results of the single correlation analysis. For OHAT, a regression model was constructed using high OHAT as the dependent variable (OHAT ≥ 5: 1, OHAT < 5: 0) to determine the amount indicating poor oral health condition. For confounding factors, number of decayed teeth and eating independence were excluded from the regression models, since those are included in the OHAT criteria or were evaluated as part of determination of long-term care need. As a result, long-term care need level, denture use frequency, and fungal amount were used as independent variables. SPSS for Windows software, ver. 25.0 (IBM SPSS, Tokyo, Japan), was used for all data analyses.

This study followed the STROBE statement for reports of observational studies (Additional file 2).

## Results

### Comparisons of health conditions and microbial amounts between participants with and without undernutrition

**Fig. 2:**
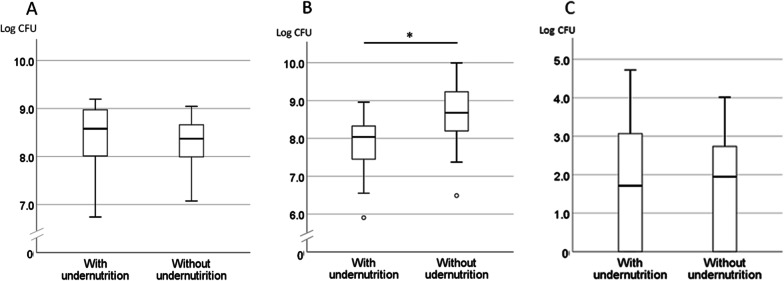
Comparisons of microbial amounts between groups with and without undernutrition. **A** Total bacteria. **B** *Streptococci*. C. Fungi. The bold line in the middle of the box indicates the median. The bottom edge of the box shows the 25th percentile and the top edge the 75th percentile. Whiskers at the bottom and top of the box indicate minimum and maximum values, respectively. Circle below the whisker indicates an outlier. *p = 0.003, t test

Fifteen of the 41 participants were determined to have undernutrition based on a BMI cut-off value of < 20, with the average ± SD for those 17.5 ± 1.43, while that was 23.5 ± 3.49 for the participants in the group without undernutrition. Other than BMI, there were no significant differences between the groups for other systemic or oral health conditions (Table 1). As for microbial measurements, only streptococcal amounts showed a statistically significant difference between groups with and without undernutrition, with the average ± SD values for streptococcal amount as the logarithm number of CFU 7.80 ± 0.86 and 8.76 ± 0.83, respectively (Fig. 2).

**Table 1:** Comparisons of health conditions and microbial amounts between participants with undernutrition and adequate nutrition

	With undernutrition (BMI ≥ 20, n = 15)	Adequate nutrition (BMI < 20, n = 26)	Total	*p* value in comparison test
BMI	17.5 ± 1.43	23.5 ± 3.49	21.3 ± 4.10	< 0.001^a^
Males/females, %	4/11, 26.7	4/22, 15.4	8/33, 19.5	0.434^b^
Age in years (average ± SD)	85.7 ± 9.70	83.9 ± 7.47	84.6 ± 8.28	0.523^a^
Care needs score (median, range)	6, 1–7	5, 1–7	5, 1–7	0.445^c^
Eating independence (not independent/independent, %)	6/9, 40.0	7/19, 26.9	13/28, 31.7	0.492^b^
Mucus score (median, range)	24.3, 0.2–28.1	25.7, 1.5–29.9	25.3, 0.2–29.9	0.315^c^
TCI score (median, range)	1, 1–9	2, 1–13	2.0, 1–13	0.173^c^
Number of present teeth (median, range)	4, 0–26	8.5, 0–25	6, 0–26	0.341^c^
Number of decayed teeth (median, range)	0, 0–4	0, 0–9	0, 0–9	0.659^c^
Number of filled teeth (median, range)	2, 0–16	6, 0–21	3, 0–21	0.108^c^
Edentulous subjects (edentulous/dentate, %)	6/9, 40.0	5/21, 19.2	11/30, 26.8	0.272^b^
Denture wearers (wearers/non-wearers, %)	8/7, 53.3	16/10, 61.5	23/18, 56.1	0.515^b^
OHAT score (median, range)	5, 1–13	5, 1–10	5, 1–13	0.512^c^

^a^t test

^b^Fisher’s exact test

^c^Mann–Whitney U test

### Comparisons of health conditions and microbial amounts between participants with and without poor oral health condition

**Fig. 3:**
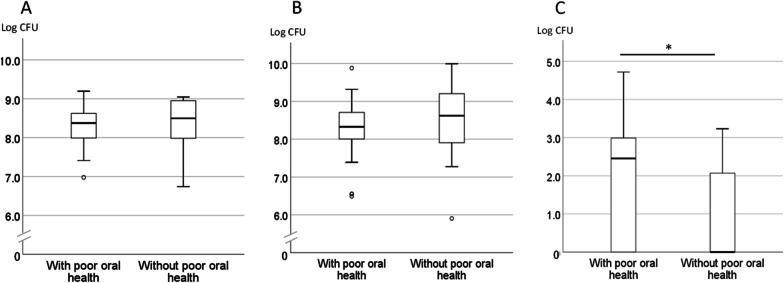
Comparisons of microbial amount between groups with and without poor oral health. **A** Total bacteria. **B** *Streptococci*. **C** Fungi. The bold line in the middle of the box indicates the median. The bottom edge of the box shows the 25th percentile and the top edge the 75th percentile. Whiskers at the bottom and top of the box indicate minimum and maximum values, respectively. Circles above and below the whiskers indicates outliers. *p = 0.028, Mann–Whitney U test

Twenty-six of the 41 participants had a high OHAT score (> 5), indicating a poor oral health condition, with the median and range for the high OHAT group 6 and 5–13, respectively, and for the low OHAT group, 2 and 1–4, respectively. As for systemic and oral health conditions, significant differences were found between the groups for care needs score, rate of participants with eating independence, and number of decayed teeth (Table 2). In microbial analyses, fungal amount was significantly higher in the high OHAT group participants with poor oral health, with median and range values of 2.45 and 0–4.72, respectively, for the group with poor oral health, and 0 and 0–3.32, respectively, for the group without poor oral health. No association with OHAT score was found for total bacterial amount or streptococcal amount (Fig. 3).

**Table 2:** Comparisons of health conditions and microbial amounts between participants with and without poor oral health condition

	With poor oral health (OHAT ≥ 5, n = 26)	Without poor oral health (OHAT < 5, n = 15)	Total^d^	*p* value in comparison test
OHAT score (median, range)	6, 5–13	2, 1–4	5, 1–13	< 0.001^c^
Males/females, %	4/22, 15.4	4/11, 26.7	8/33, 19.5	0.434^b^
Age in years (average ± SD)	85.9 ± 8.49	82.3 ± 7.62	84.6 ± 8.28	0.181^a^
BMI	21.2 ± 4.65	21.5 ± 3.04	21.3 ± 4.10	0.812^a^
Care needs score (median, range)	6, 2–7	1, 1–6	5, 1–7	< 0.001^c^
Eating independence (not independent/independent, %)	13/13, 50.0	0/15, 0.0	13/28, 46.4	0.001^b^
Mucus score (median, range)	24.1, 0.2–29.9	26.5, 18.8–29.9	25.3, 0.2–29.9	0.181^c^
TCI socre (median, range)	2, 1–13	2, 1–9	2.0, 1–13	0.825^c^
Number of present teeth (median, range)	7, 0–26	2, 0–23	6, 0–26	0.583^c^
Number of decayed teeth (median, range)	0, 0–9	0, 0–1	0, 0–9	0.030^c^
Number of filled teeth (median, range)	4.5, 0–19	2, 0–21	3, 0–21	0.947^c^
Edentulous subjects (edentulous/dentate, %)	5/21, 23.8	6/9, 40.0	11/30, 73.2	0.272^b^
Denture wearer (wearers/non-wearers, %)	12/14, 46.2	11/4, 73.3	23/18, 56.1	0.114^b^

^a^t test

^b^Fisher’s exact test

^c^Mann–Whitney U test

^d^Same data as shown in Table 1 excluding OHAT

### Measurements related with microbial amounts

**Fig. 4:**
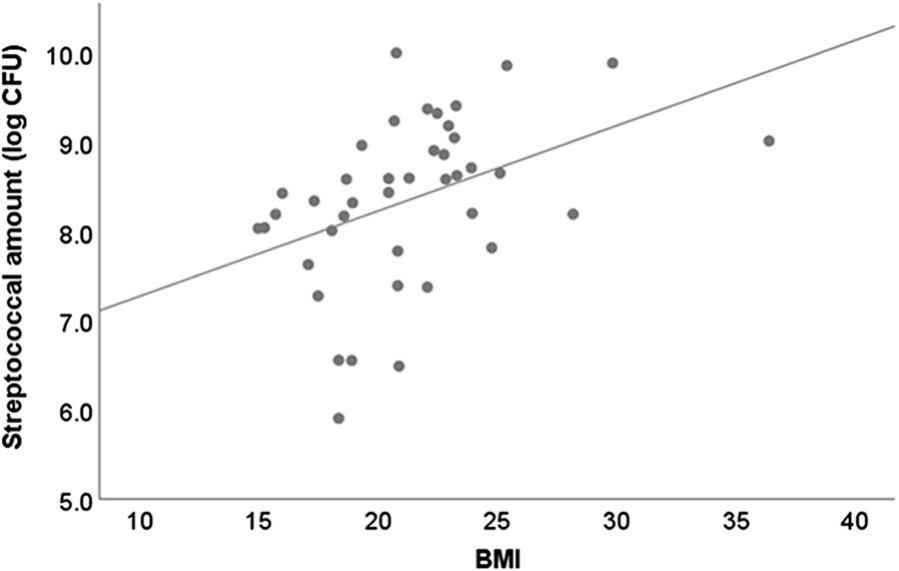
Scatter plot showing correlation between streptococcal amount and BMI. Straight line indicates regression (r = 0.420)

The association between each microbial amount and other observed values was assessed by single correlation coefficient analysis. Table 3 shows correlation coefficients of significantly related measurements with the amount of any microorganism in addition to OHAT score. In the examination of correlations between microbial amounts, there was a significant positive relationship between total bacterial and streptococcal amounts (r = 0.593, *p* < 0.001). On the other hand, fungi showed a negative relationship with both total bacteria and streptococci, though that was slight and not significant. For nutritional condition, only streptococcal amount showed a significant correlation with BMI (r = 0.420, *p* = 0.006). Linear correlations are shown in Fig. 4. TCI was positively related with total bacterial amount (ρ = 0.346, *p* = 0.029) and negatively related with fungal amount (ρ = − 0.331, *p* = 0.037). Moreover, fungal amount showed a significant negative correlation with Mucus score (ρ = − 0.383, *p* = 0.018). OHAT score did not have a significant relationship with fungal amount (ρ = − 0.287, *p* = 0.068).

**Table 3:** Correlations among microbial amounts, and nutritional and oral conditions

	Total bacterial amount	Streptococc-al amount	Fungal amount	BMI	Eating independence^b^	TCI	Mucus score	OHAT score
Total bacterial amount	1.00	0.593^a^	− 0.205	− 0.104^a^	0.150	0.346	− 0.011	0.073
		< 0.001	0.199	0.516	0.348	0.029	0.949	0.651
Streptococcal amount	0.593^a^	1.00	− 0.141	0.420^a^	− 0.290	− 0.053	0.075	− 0.171
	< 0.001		0.379	0.006	0.066	0.746	0.654	0.286
Fungal amount	− 0.205	− 0.141	1.00	− 0.055	0.228	− 0.331	− 0.383	0.287
	0.199	0.379		0.732	0.152	0.037	0.018	0.068
BMI	− 0.104^a^	0.420^a^	− 0.055	1.00	− 0.269	− 0.244	0.017	− 0.184
	0.516	0.006	0.732		0.089	0.130	0.921	0.250
Eating independence	0.150	− 0.290	0.228	− 0.269	1.00	0.169	− 0.157	0.565
	0.348	0.066	0.152	0.089		0.297	0.346	− < 0.001
TCI	0.346	− 0.053	− 0.331	− 0.244	0.169	1.00	0.091	0.010
	0.029	0.746	0.037	0.130	0.297		0.585	0.952
Mucus score	− 0.011	0.075	− 0.383	0.017	− 0.157	0.091	1.00	− 0.191
	0.949	0.654	0.018	0.921	0.346	0.585		0.250
OHAT score	0.073	− 0.171	0.287	− 0.184	0.565	0.010	− 0.191	1.00
	0.651	0.286	0.068	0.250	< 0.001	0.952	0.250	

Values significantly related with amount of microorganisms and OHAT are shown

Upper value shows correlation coefficient and lower value *p* value

^a^Pearson’s correlation coefficient. The other correlation coefficient was determined as Spearman’s rank correlation coefficient

^b^Degree of eating independence was scored as follows: 1, independent; 2, partial assistance needed; 3, full assistance needed

### Multiple logistic regression analyses

In the full model with "not undernutrition" as the dependent variable, streptococcal amount was the only significant variable, with an adjusted odds ratio (AOR) of 6.10 (*p* = 0.006). After a stepwise procedure, streptococcal amount was selected for the final model (Table 4). As a result, the adjusted odds ratio (AOR) was 6.32, with a 95% confidential interval (CI) of 1.71–23.3 (*p* = 0.006) (Table 5).

**Table 4:** Logistic regression models for poor oral health condition

	Variable	AOR	95% CI	*p* value
Full enter model	Long-term care need level	2.60	1.40–4.82	0.002
	Fungal amount	2.72	1.05–7.05	0.039
	Denture use frequency	0.386	0.107–1.40	0.147
Final model after stepwise procedure	Long-term care need level	2.60	1.52–5.13	0.002
	Fungal amount	2.44	0.987–6.04	0.053

Dependent variable: OHAT > 5: 1, OHAT ≤ 5: 0

AOR, adjusted odds ratio; CI, confidential interval

**Table 5:** Logistic regression models for adequate nutrition

	Variable	AOR	95% CI	*p* value
Full enter model	Streptococcal amount	6.10	1.66–22.4	0.006
	TCI	0.816	0.620–1.07	0.146
	Eating independence	0.744	0.264–2.10	0.577
Final model after stepwise procedure	Streptococcal amount	6.32	1.71–23.3	0.006
	TCI	0.808	0.620–1.05	0.114

Dependent variable: BMI ≥ 20: 1, BMI < 20: 0

AOR, adjusted odds ratio; CI, confidential interval

In multiple regression analyses related to high OHAT (poor oral health condition), long term care need level and fungal amount were shown to be significant variables in the full enter model (AOR: 2.60 and 2.72, *p* = 0.002 and 0.39, respectively). After a stepwise procedure, long-term care need level and fungal amount were selected for the final model (Table 6). For fungal amount, the AOR was 2.44, with a 95% CI of 0.987–6.04 (*p* = 0.053).

## Discussion

Among the elders participated in this study, the amount of oral streptococci was found to be significantly lower in the undernutrition group (7.80 ± 0.86 vs. 8.67 ± 0.83), while a significant association (r = 0.420) between amount of streptococci and BMI was found in the entire participants. Furthermore, using a multiple logistic regression model in which “not malnutrition” was the dependent variable, the amount of oral streptococci was selected as a significant independent variable with high AOR (6.32) in the final model after a stepwise procedure. BMI, which is widely used to determine whether an individual is under- or overweight, and also employed in a variety of nutrition assessment tools as a criterion for assessing undernutrition [41, 43, 44], was used in this study as an indicator of systemic nutritional condition. The average BMI value for the present participants was 21.3, within the average range (20.6–22.5) reported in previous investigations conducted in Japan of institutionalized older individuals [45–47]. Thus, their nutritional status was considered to be comparable to that of institutionalized elderly in Japan.

Bacteria belonging to the genus *Streptococcus* are the initial inhabitants of the oral cavity, as they are generally acquired right after birth and play an important role in assembly of the oral microbiota [14]. Moreover, dominance continues until old age [48]. Most oral streptococci are not pathogenic and thought to contribute to oral homeostasis [14], though a change in eating conditions could have an influence on the normal *Streptococcus*-dominant balance of the oral microbiota. Takeshita et al. reported that dominant genera such as *Streptococcus* were observed in much lower proportions in tube-fed subjects as compared to those fed orally [49]. Even when an oral-environmental change is not as drastic as tube feeding, a decrease in food intake could have a negative effect on growth of oral streptococci, since eating by mouth supplies nutrition to microbes necessary for maintenance of the indigenous oral microbiota. Staple foods in most human populations include starch rich crops such as rice, wheat, and potatoes. Polysaccharides composing starch are degraded by cooking with heat and salivary amylase into mono- or disaccharides, which are easily metabolized by oral streptococci [14, 50, 51]. A decrease in the amount of food intake via the oral cavity restricts the energy source for streptococcal organisms as well as the human host. Such restriction results in energy source reduction as well as aging-related changes in the oral cavity, represented by decreased saliva flow and an altered oral immune system [52]. Saliva contains secretory IgAs and anti-microbial peptides. However, commensal oral streptococci, such as *S. mitis, S. oralis*, and *S. sanguinis*, have become adapted to those components [53–55]. Thus, a decrease in saliva may cause *Streptococcus* to fail to dominate the oral cavity, consequently allowing easier grow for other microorganisms.

On the other hand, fungal amount is related with oral moisture condition and oral health. Wu et al. reported that oral care intervention in patients with end stage of cancer reduced detection of *Candida* on the tongue, accompanied by a decrease in OHAT score [56], which was confirmed by the present results. OHAT criteria for assessments of lip, tongue, gum tissues, and saliva include dryness, with oral dryness one of the significant factors related to fungal colonization in the oral cavity [57, 58]. Saliva contains glycoproteins, which act to inhibit adhesion of fungi to epithelial cells [59], and also antimicrobial proteins, histatins, defensins, and other components that can suppress *Candida* spp. [60]. Thus, a dry condition is preferable for fungi colonization. In contrast to streptococci, fungi have an ability to grow under altered mucosal conditions. Such differences in suitable conditions for colonization may allow fungi and streptococci to grow competitively in the oral cavity. However, previous in vitro studies of the relationship between fungal and streptococcal colonization have shown contradictory results. Some demonstrated that oral streptococci contribute to colonization of oral fungi or modification of their virulence [61, 62], while others have reported that a specific streptococcus could inhibit *Candida* colonization in the oral cavity [63–65]. In the present study, both fungal and streptococcal amounts in the oral cavities of the same subjects were determined, and the results showed a negative, though not significant, relationship between oral streptococcal and fungal abundance. The actual balance of fungi and streptococcus colonization in the human oral cavity is unknown, and the present results as well did not show an obvious relationship. Elucidation of the prevalence of both oral fungi and indigenous streptococci in the oral cavity is important for understanding the relationship between frailty and oral health in elderly individuals. It is considered that the oral streptococcus-specific primer established in the present study will be an effective tool for future surveys.

This study has some limitations. First, it is difficult to generalize the results due to the small number of participants residing at a single institution. Also, direct assessments of symptoms of frailty and amount of food intake were not performed, thus the actual relationships of oral microbiota with frailty or food intake amount were not clearly elucidated. Furthermore, because of the cross-sectional design, changes in BMI were not recorded, though it is known that a rapid decrease in BMI is as important as a stable state for assessment of frailty in elders [37]. Additionally, some potential biases are assumed. For example, this study showed that OHAT values were lower and oral health better in participants who were able to eat independently. However, dietary management for elder individuals by a caregiver may result in stabilization of food intake and help the individual maintain a better BMI as compared to those who eat independently based on their own preferences. Thus, additional studies with greater numbers of subjects and multiple institutions that utilize longitudinal observations are required.

Based on the results of this study, in order to prevent progression of frailty in elder individuals, greater consumption of foods with moisturizing effects may be effective to maintain a balance of streptococci and fungi that results in a streptococcus-dominant state. In the future, it will be desirable to develop probiotic foods that inhibit fungi and promote streptococcal growth. As an indicator of the effects of such interventions, simultaneous evaluation of streptococcus and fungus amounts is useful.

## Conclusion

Oral streptococcal levels were found to be associated with the general nutritional status of institutionalized older individuals, while oral fungal levels were associated with oral health status. Therefore, it is necessary to survey both oral indigenous streptococci as well as fungi to understand the relationship of frailty with oral microbiota, as well as assess the effects of intervention on the oral health of elderly individuals.

## Supplementary Information

**Additional file 1.** PCR products from 16 strains of streptococcus specific primer designed for this study.

**Additional file 2.** Strobe check list.

## Data Availability

The primer sequences targeting all streptococci created for this study have not been uploaded. Datasets used and/or analyzed in this study are available from the corresponding author upon reasonable request.

## Abbreviations

AOR: Adjusted odds ratio
BMI: Body Mass Index
CFU: Colony forming units/mL
CI: Confidential interval
OHAT: Oral health assessment tool
SD: Standard deviation
TCI: Tongue Coating Index
WHO: World Health Organization
