# Supplementary material for: Associations of streptococci and fungi amounts in the oral cavity with nutritional and oral health status in institutionalized elders: a cross sectional study
Source: BMC Oral Health. 2021 Nov 19;21:590. doi: 10.1186/s12903-021-01926-0 (PMC8603531; doi:10.1186/s12903-021-01926-0)
Supplement: Supplementary file 1 — Additional file 1. PCR products from 16 strains of streptococcus specific primer designed for this study. [file 12903_2021_1926_MOESM1_ESM.docx]

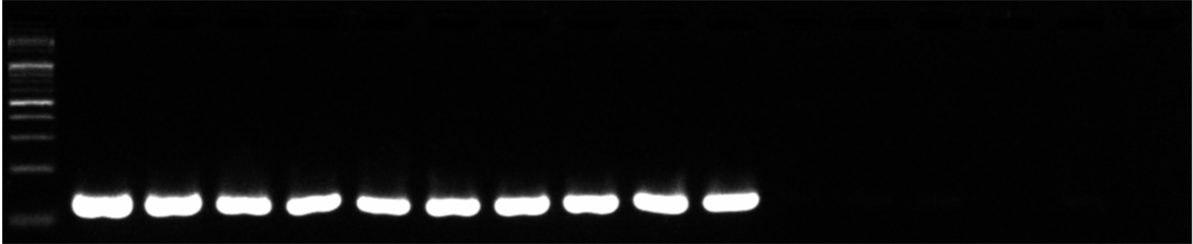


M 1 2 3 4 5 6 7 8 9 10 11 12 13 14 15 16

9 *S. constellatus* ATCC 27823

10 *S. anginosus* NCTC10713

11 *Staphylococcus aureus* ATCC 25923

12 *Staphylococcus epidermidis* ATCC 12228

13 *Streptococcus pyogenes* ATCC 12334

14 *Enterococcus faecalis* ATCC 29212

15 *Fusobacterium nucleatum* ATCC 25586

16 *Escherichia coli* BL21

M： marker

1 *Streptococcus mutans* ATCC 25175

2 *S. sobrinus* ATCC 27351

3 *S. salivarius* ATCC 7073

4 *S. oralis* ATCC 10557

5 *S. gordonii* ATCC 10558

6 *S. sanguinis* ATCC 10556

7 *S. mitis* ATCC 49456

8 *S. intermedius* GAI 1157
