# Supplementary material for: Associations of streptococci and fungi amounts in the oral cavity with nutritional and oral health status in institutionalized elders: a cross sectional study
Source: BMC Oral Health. 2021 Nov 19;21:590. doi: 10.1186/s12903-021-01926-0 (PMC8603531; doi:10.1186/s12903-021-01926-0)
Supplement: Supplementary file 2 — Additional file 2. Strobe check list. [file 12903_2021_1926_MOESM2_ESM.docx]

STROBE Statement—checklist of items that should be included in reports of observational studies

|  | **Item No.** | **Recommendation** | **Page No.** | **Relevant text from manuscript** |
| --- | --- | --- | --- | --- |
| **Title and abstract** | 1 | (*a*) Indicate the study’s design with a commonly used term in the title or the abstract | 1 | a cross sectional study |
|  |  | (*b*) Provide in the abstract an informative and balanced summary of what was done and what was found | 2-3 | to elucidate the associations of nutritional and oral health conditions with prevalence of bacteria and fungi in the oral cavity of older individuals.  Oral streptococci were found to be associated with systemic nutritional condition and oral fungi with oral health condition. |
| **Introduction** |  |  |  |  |
| Background/rationale | 2 | Explain the scientific background and rationale for the investigation being reported | 4 | deterioration of general health and nutritional status may disrupt the normal microbiota by increasing fungi and decreasing indigenous bacteria amounts. |
| Objectives | 3 | State specific objectives, including any prespecified hypotheses | 5 | We speculated that simultaneous measurements of oral fungal and streptococcus levels would be useful for determining systemic and oral frailty. |
| **Methods** |  |  |  |  |
| Study design | 4 | Present key elements of study design early in the paper | 6 | This was a cross-sectional observational study |
| Setting | 5 | Describe the setting, locations, and relevant dates, including periods of recruitment, exposure,  follow-up, and data collection | 6 | conducted at a nursing institution located in Iwate Prefecture, Japan. |
| Participants | 6 | (*a*) *Cohort study*—Give the eligibility criteria, and the sources and methods of selection of participants. Describe methods of follow-up  *Case-control study*—Give the eligibility criteria, and the sources and methods of case ascertainment and control selection. Give the rationale for the choice of cases and controls *Cross-sectional study*—Give the eligibility criteria, and the sources and methods of selection of  participants | 6 | Exclusion criteria included administration of antimicrobial or antifungal drugs within one month of the survey, as well as current status of undergoing parenteral nutrition. |
|  |  | (*b*) *Cohort study*—For matched studies, give matching criteria and number of exposed and unexposed  *Case-control study*—For matched studies, give matching criteria and the number of controls per  case |  | Not applicable |
| Variables | 7 | Clearly define all outcomes, exposures, predictors, potential confounders, and effect modifiers.  Give diagnostic criteria, if applicable | 7, 8 | The primary outcomes were amounts of oral streptococci, oral fungi, and total bacteria, which were determined using a quantitative polymerase chain reaction (PCR) method.  information regarding potential confounders or effect modifiers, including gender,…. was provided by the institution. |
| Data sources/  measurement | 8* | For each variable of interest, give sources of data and details of methods of assessment  (measurement). Describe comparability of assessment methods if there is more than one group | 8-12 | Present tooth status including decayed and filled teeth was assessed according to the WHO criteria.  Microbial samples were collected immediately after the oral examination  A specific primer for amplifying the genome of oral streptococci was designed based on the *S. mutans* ATCC 25175 gene (NCBI Accession No. EF536028)  PCR assays were performed using a Thermal Cycler Dice Real-Time System II |
| Bias | 9 | Describe any efforts to address potential sources of bias | 8 | Differences in dietary habits were not considered in this study, because the facility provides nearly the same food menu to all residents for each meal service.Potential confounders or effect modifiers concerning oral health were assessed by oral examinations performed by two well-calibrated dentists |
| Study size | 10 | Explain how the study size was arrived at | 6 | Informed consent was obtained from 42 of the 160 residents. |
| Quantitative variables | 11 | Explain how quantitative variables were handled in the analyses. If applicable, describe which groupings were chosen and why | 12, 13 | Age, BMI, care needs score, number of teeth, TCI, Mucus score, OHAT score, and amounts of oral microorganisms were used as continuous variables.  participants were classified as BMI <20 (undernutrition) or ≥20 (adequate nutrition) |
| Statistical methods | 12 | (*a*) Describe all statistical methods, including those used to control for confounding | 12, 13 | Single correlations for combinations of quantitative variables were tested using Pearson’s correlation coefficient analysis, while Spearman's rank correlation analysis was used for combinations of rank or categorical variables after the categorical variables were transformed into binary variables. |
|  |  | (b) Describe any methods used to examine subgroups and interactions | 12, 13 | Mann-Whitney’s U test was used for rank variables and Fisher’s exact test for categorical variables |
|  |  | (c) Explain how missing data were addressed | Not applicable |  |
|  |  | (d)*Cohort study*—If applicable, explain how loss to follow-up was addressed  *Case-control study*—If applicable, explain how matching of cases and controls was addressed *Cross-sectional study*—If applicable, describe analytical methods taking account of sampling | Not applicable |  |
|  |  | (e) Describe any sensitivity analyses | Not applicable | e |
| **Results** |  |  |  |  |
| Participants | 13* | (a) Report numbers of individuals at each stage of study – eg numbers potentially eligible, examined for eligibility, confirmed eligible, included in the study, completing follow-up, and analysed | 6 | Consequently, 41 elder individuals (8 males, 33 females) with an average age ± standard deviation (SD) of 84.6 ± 8.3 years (range 70-105 years) completed the present study protocol |
|  |  | (b) Give reasons for non-participation at each stage | 6 | one rejected participation, after which none withdrew their consent. |
|  |  | (c) Consider use of a flow diagram | Figure 1 | We used a flow diagram. |
| Descriptive data | 14* | (a) Give characteristics of study participants (eg, demographic, clinical, social) and information on exposure and potential confounders. | Table 1, 2  23 | dietary management for elder individuals by a caregiver may result in stabilization of food intake and help the individual maintain a better BMI as compared to those who eat independently based on their own preferences. |
|  |  | (b) Indicate number of participants with missing data for each variable of interest | Not applicable |  |
|  |  | (c) *Cohort stud*y-Summarise follow-up time (eg, average and total amount) | Not applicable |  |
| Outcome data* | 15* | *Cohort study*—Report numbers of outcome events or summary measures over time | Not applicable |  |
|  |  | *Case-control study—*Report numbers in each exposure category, or summary measures of exposure | Not applicable |  |
|  |  | *Cross-sectional study—*Report numbers of outcome events or summary measures | Table 1, 2 |  |
| Main results | 16 | (a) Give unadjusted estimates and, if applicable, confounder-adjusted estimates and their precision (eg, 95% confidence interval). Make clear which continuous variables were categorized. | Table 4, 5 |  |
|  |  | (b) Report category boundaries when continuous variables were categorized | 12-13 | the participants were classified as BMI <20 (undernutrition) or ≥20 (adequate nutrition), according to the criteria for Asian elderly aged 70 years or older |
|  |  | (c) If relevant, consider translating estimates of relative risk into absolute risk for a meaningful time period. | Table 4, 5  19-20 | streptococcal amount was the only significant variable, with an adjusted odds ratio (AOR) of 6.10 (*p* = 0.006). |
| Other analyses | 17 | Report other analyses done—eg analyses of subgroups and interactions, and sensitivity analyses | Figure 4 |  |
| **Discussion** |  |  |  |  |
| Key results | 18 | Summarise key results with reference to study objective | 21 | Among the elders participated in this study, the amount of oral streptococci was found to be significantly lower in the undernutrition group (7.80±0.86 vs. 8.67±0.83), while a significant association (r=0.420) between amount of streptococci and BMI was found in the entire participants. |
| Limitations | 19 | Discuss limitations of the study, taking into account sources of potential bias or imprecision. Discuss  both direction and magnitude of any potential bias | 23 | This study has some limitations. First, it is difficult to generalize the results due to the small number of participants residing at a single institution. |
| Interpretation | 20 | Give a cautious overall interpretation of results considering objectives, limitations, multiplicity of analyses, results from similar studies, and other relevant evidence | 23 | Based on the results of this study, in order to prevent progression of frailty in elder individuals, greater consumption of foods with moisturizing effects may be effective to maintain a balance of streptococci and fungi that results in a streptococcus-dominant state. |
| Generalisability | 21 | Discuss the generalisability (external validity) of the study results | 23 | it is difficult to generalize the results due to the small number of participants residing at a single institution. |
| **Other information** |  |  |  |  |
| Funding | 22 | Give the source of funding and the role of the funders for the present study and, if applicable, for the  original study on which the present article is based | 25 | A research grant from Iwate Prefecture for Strategic Developmental Research was received for this study. |

*Give information separately for cases and controls in case-control studies and, if applicable, for exposed and unexposed groups in cohort and cross-sectional studies.

**Note:** An Explanation and Elaboration article discusses each checklist item and gives methodological background and published examples of transparent reporting. The STROBE checklist is best used in conjunction with this article (freely available on the Web sites of PLoS Medicine at [http://www.plosmedicine.org/,](http://www.plosmedicine.org/) Annals of Internal Medicine at [http://www.annals.org/,](http://www.annals.org/) and Epidemiology at [http://www.epidem.com/).](http://www.epidem.com/)) Information on the STROBE Initiative is available at [www.strobe-statement.org.](http://www.strobe-statement.org/)
